# First report of X-linked hypohidrotic ectodermal dysplasia with a hemizygous c.1142G > C in the EDA gene: variant of uncertain significance or new pathogenic variant?

**DOI:** 10.1186/s13052-021-01078-5

**Published:** 2021-06-02

**Authors:** Mario Tumminello, Antonella Gangemi, Federico Matina, Melania Guardino, Bianca Lea Giuffrè, Giovanni Corsello

**Affiliations:** 1 Neonatal Intensive Care Unit, Villa Sofia-Cervello Hospital, Palermo, Italy; 2 Department of Sciences for Health Promotion and Mother and Child Care “G. D’Alessandro”, University of Palermo, Palermo, Italy

**Keywords:** Hypoidrotic ectodermal dysplasia, X-linked, EDA gene, Variants of uncertain significance (VUS)

## Abstract

**Background:**

Hypohidrotic Ectodermal Dysplasia (HED) is a genetic disorder which affects structures of ectodermal origin. X-linked hypohidrotic ectodermal dysplasia (XLHED) is the most common form of disease. XLHED is characterized by hypotrichosis, hypohydrosis and hypodontia. The cardinal features of classic HED become obvious during childhood.

Identification of a hemizygous EDA pathogenic variant in an affected male confirms the diagnosis.

**Case presentation:**

We report on a male newborn with the main clinical characteristics of the X-linked HED including hypotrichosis, hypodontia and hypohidrosis. Gene panel sequencing identified a new hemizygous missense variant of uncertain significance (VUS) c.1142G > C (p.Gly381Ala) in the EDA gene, located on the X chromosome and inherited from the healthy mother.

**Conclusion:**

Despite the potential functional impact of VUS remains uncharacterized, our goal is to evaluate the clinical potential consequences of missense VUS on EDA gene. Even if the proband’s phenotype is characteristic for classic HED, further reports of patients with same clinical phenotype and the same genomic variant are needed to consider this novel VUS as responsible for the development of HED.

## Background

Ectodermal dysplasia (ED) includes a large and heterogeneous group of rare congenital conditions with structural and functional abnormalities in various tissues originating from the ectodermal layer of the developing embryo. Pinheiro and Freire-Maia defined as ED any condition with lack or dysgenesis of at least two of the ectodermal derivatives: hair, nails, teeth or eccrine sweet glands [1, 2]. The birth prevalence is estimated around 7 cases in 10,000 live births [1, 3].

The two most common forms of the disease are hypohidrotic and anhidrotic ED [4, 5]. Hypohidrotic ectodermal dysplasia (HED) is characterized by a triad of signs comprising sparse hair (hypotrichosis), abnormal or missing teeth (anodontia or hypodontia), and inability to sweat (anhidrosis or hypohidrosis) [6, 7].

Typical clinical manifestations also include dryness of the skin, eyes, airways, and mucous membranes presumably due to the defective development of several exocrine glands. HED can be associated with dysmorphic features (forehead bumps, rings under the eyes, everted nose and prominent lips) and occasionally with absent nipples and weight deficits [8, 9].

Hypohidrotic ED may be inherited as X-linked, autosomal dominant, and autosomal recessive patterns. Four genes (EDA, EDAR, EDARADD, WNT10A) account for 90% of hypoidrotic/anhidrotic ectodermal dysplasia cases [10, 11]. X-linked hypohidrotic ectodermal dysplasia (XLHED) is the most common form of disease. The EDA gene, located at Xq12-q13.1 and encoding the transmembrane type II ectodysplasin-A (EDA) protein, which belongs to the tumor necrosis factor superfamily, is responsible for XLHED through EDA-EDAR (EDA receptor)-EDARADD (EDAR-associated death domain) pathway [12, 13]..

The function of EDA protein in pathways regulating ectodermal development, is a key regulator of hair follicle and sweat gland initiation [13, 14]. Various mutations in EDA account for vast majority of XLHED cases. The most frequent mutation type is the missense/nonsense [12, 15].

We report on a three-month-old boy with XLHED showing a novel hemizygous missense variant of uncertain significance (VUS) in the EDA gene, detected by Next Generation Sequencing (NGS).

## Case report

A three-month-old, caucasian boy infant was referred to our observation due to decreased sweating, dry skin and absence of hair on the scalp. Family history for inherited diseases was unremarkable. The facial features, hair, teeth, skin and nails of the mother appeared to be normal. He was the second child of healthy non-consaguineous parents. The first child was a healthy female.

He was born by spontaneous delivery after normal pregnancy, with no birth-related and perinatal complications. Apgar scores were 8 and 9 at 1 and 5 min, respectively. Birth weight was 2390 g (4th centile), length 47 cm (12th centile), and head circumference 33 cm (19th centile).

**Fig. 1:**
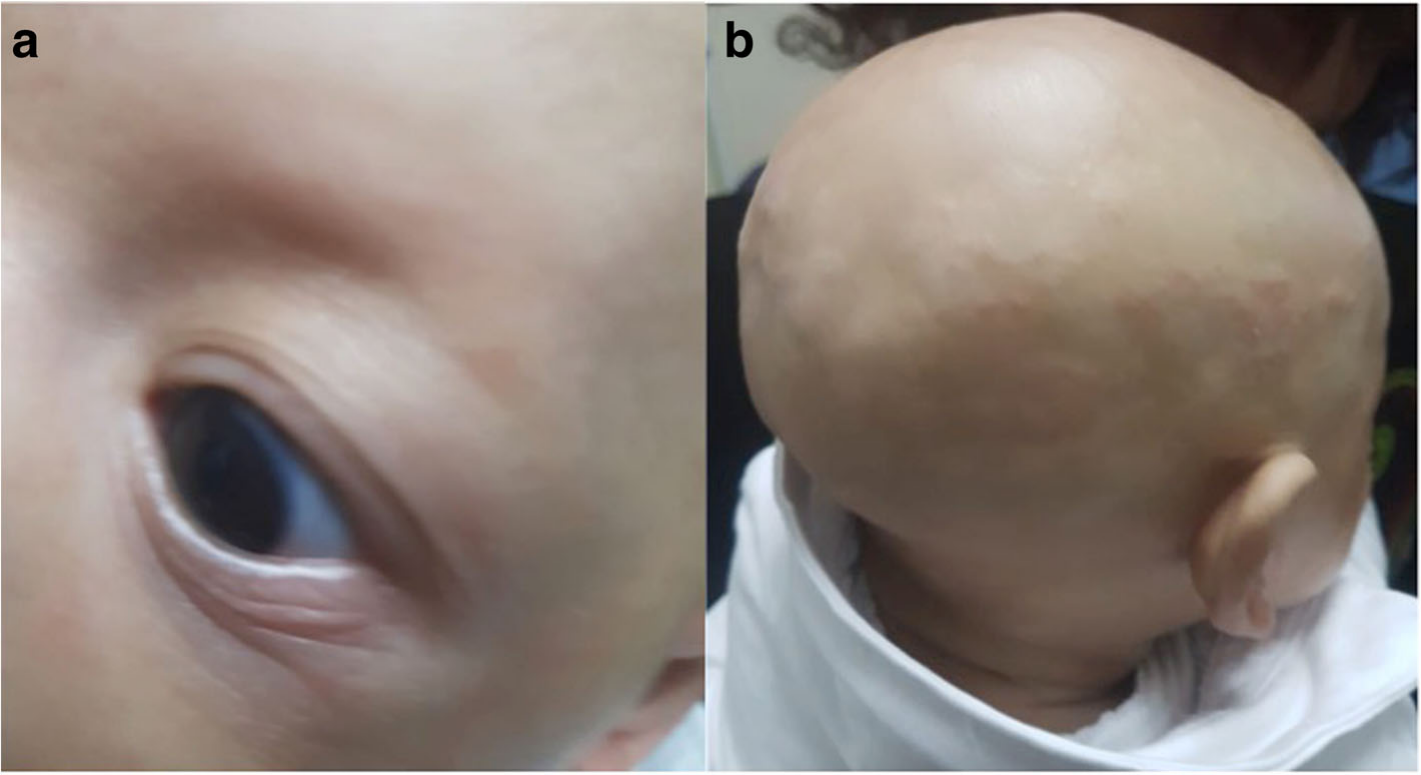
X-linked hypohidrotic ectodermal dysplasia. **a** missing eyebrows, sparse eyelashes; (**b**) scaphocephaly, missing hair, eczematous dermatitis.

Physical examination showed scaphocephaly, prominent forehead, forehead bumps, rings under the eyes, hypertelorism, epicanthic fold, everted nose, depressed nasal bridge and prominent lips. Both the upper and lower eyelids showed sparse eyelashes. The hair on the scalp and the eyebrows were absent. The skin was thin, pale, dry and exfoliating with eczematous dermatitis widespread, especially in the scalp. The skin from around the eyes and mouth showed linear wrinkled and was hyperpigmented (Fig. 1). The parents reported dryness of eyes. Neuromotor and mental development index score of Bayley II scale were normal.

Abdominal and cerebral ultrasound screening did not detect anomalies and routine ECG and echocardiogram evaluation were normal.

Molecular genetic studies were performed on genomic DNA extracted from peripheric blood, through Next Generation Sequencing (NGS). A multigene Ectodermal dysplasia panel, that included EDA, EDAR, EDARADD, EDA2R, TRAF6, NFKBIA, CDH3, WNT10A, was performed. The analysis revealed the hemizygous variant, NM_001399.4: c.1142G > C (p.Gly381Ala) in EDA gene located on the X chromosome. The variant was tested for familial segregation showing heterozygous maternal state. The mother did not show any sign of HED.

**Fig. 2:**
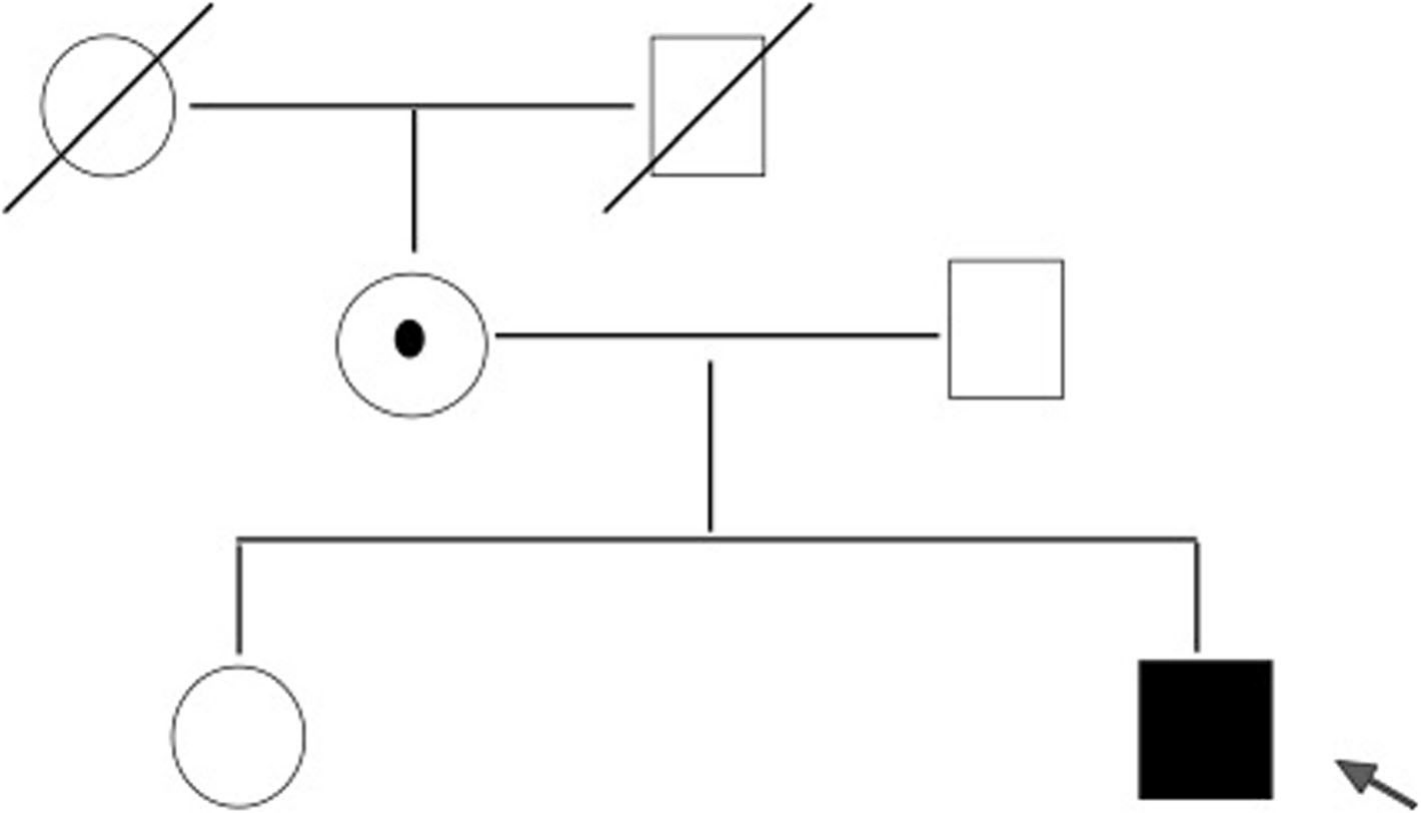
Pedigree of the patient

The same variant was not found in the father of the proband and the sibling. Pedigree analysis showed an X-linked pattern of inheritance (Fig. 2).

We analyzed the variant in silico by using software Scale-Invariant Feature Transform (SIFT), DANN, DEOGEN, MutationTaster, for the prediction of possible deleterious variants in human proteins. Combined Annotation Dependent Depletion (CADD), another algorithm used to help assess the potential pathogenicity of a variant, display scores for this variant of 26,9.

We established a multidisciplinary follow-up: ophtalmologic evaluation, hearing screen and visual evoked potentials were normal at 5 months.

The following physical examinations showed delayed teeth eruption: only two small, conical canines were present at 15 months.

The last clinically evaluation was performed at 24 months of age, with persisting poor weight gain (3th centile), reduced ability to sweat, heat intolerance, chronic eczematous rash especially on the face, wrinkled periorbital skin with Dennie-Morgan infraorbital fold.

**Fig. 3:**
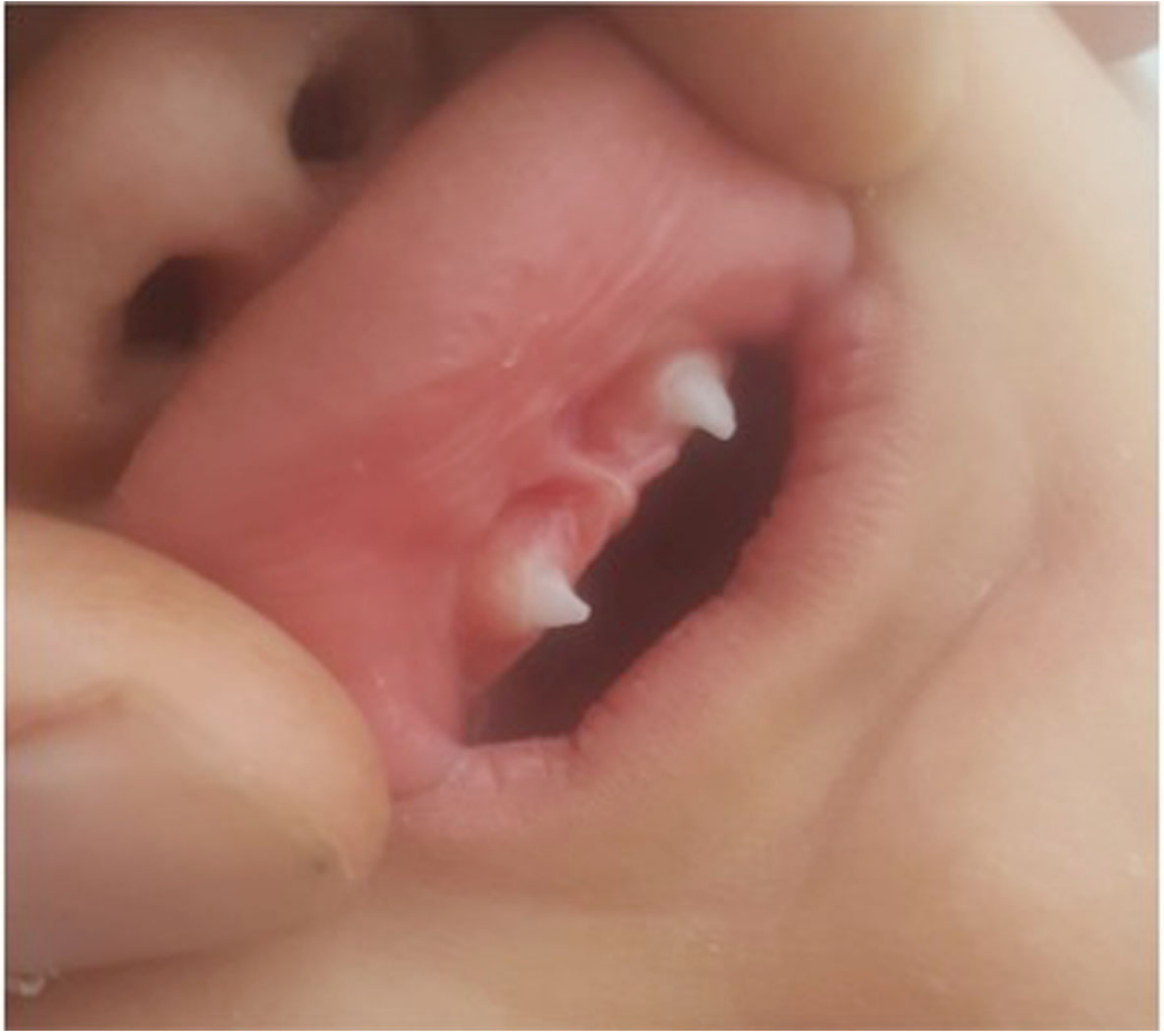
Severe oligodontia. Two conical anterior teeth and a wide midline diastema

Intraoral examination revealed severe oligodontia with only two conical anterior teeth eruption previously described and a wide midline diastema (Fig. 3).

Orthodontic management was planned in order to improve the limited chewing function ability.

Neuropsychomotor follow-up and mental development index score of Bayley II scale were in the normal range for the age.

## Discussion and conclusions

The molecular pathogenesis of HED is not yet fully understood. *EDA* is the gene responsible for X-linked HED [6]. Classic HED is often diagnosed after infancy in patients showing typical hypotrichosis, hypohidrosis, and hypodontia. In the male proband the diagnosis of classic HED was established with the above characteristic features.

Growth abnormalities, measured as weight deficits, were present at an early age in children with XLHED syndromes, as observed in our patient, and persisted through adolescence [16]. Causal factors that result in these abnormal patterns of growth are unknown. The oligodontia, in addition to salivary gland dysfunction, may prevent adequate caloric intake and may be one of probably multiple causes of impaired weight gain [17, 18].

The identification of a hemizygous EDA pathogenic variant or biallelic *EDAR*, *EDARADD* or *WNT10A* pathogenic variants confirmed the diagnosis [6, 19].

EDA mutations had been considered as genetic conditions, without a clear genotype-phenotype correlation [15, 20]. Burger and Schneider (2014) suggested a reproducible association of common EDA genotypes with XLHED phenotypes. A systematic mapping of EDA mutations with an evaluation of quantifiable clinical data may help to distinguish the pathogenetic gene mutations with respect to those allowing a residual ectodysplasin A activity [15, 21]. A multigene panel also including *EDA*, *EDAR*, *EDARADD*, *WNT10A* may be useful for the differential diagnosis.

Different pathogenic variants have been identified in *EDA* [19, 22]*.* The missense variant reported in our study is novel and does not result among those reported in the International literature (PubMed/Medline), in the Catalog of Human Genes and Genetic Disorders (OMIM), in the Genome Aggregation Database (GnomAD), ClinVar and in The Human Gene Mutation Database.

According to the American College of Medical Genetics and Genomics this genetic variant is currently considered VUS.

VUS may or may not be disease-causing or associated with increased risk of an abnormal phenotype; the identification of a variant of uncertain significance does not confirm or exclude a diagnosis. A VUS does not meet the criteria to be classified as pathogenic or benign [23].

Despite the variant reported in our study has been described as VUS with an imprecise potential functional impact, the prediction tools used suggest a potential pathogenetic effect.

To our knowledge, this is the first report of a VUS in a XLHED and it may be demonstrate that a mutation of the EDA gene caused by a hemizygous variant, c.1142G > C on the X chromosome, can lead to the XLHED clinical spectrum. Therefore, in consideration of the proband phenotype, a causative role of the variant found in hemizygosis in the gene EDA cannot be excluded.

Furthermore, only male hemizygotes are affected by this X-linked disease, being the heterozygous females clinically normal, as it happens in the vast majority of XLHED cases accounting for gene mutation.

In conclusion, despite we know many genetic variations involved in the development of HED, the latest investigation techniques such as NGS could increase our knowledge about genetic etiology.

We deemed it useful to describe and characterize a novel variant of EDA because we hope that a great number of further studies on the effect of normal and abnormal function of EDA protein will lead to a better knowledge of this group of ED.

Even if the proband’s phenotype is characteristic for classic HED, further reports of patients with same clinical phenotype and the same genomic variant are needed to consider this novel VUS as responsible for the development of HED.

If confirmed by other reports, this novel c.1142G > C (p.Gly381Ala) variant could broaden the spectrum of genes mutations involved in the development of HED.

Future studies are mandatory to elucidate genomic and epigenomic susceptibility factors, which could cause mutations on EDA gene.

## Data Availability

The clinical data used during the current report are available from the corresponding author on reasonable request.
